# Supplementary material for: Predicting visual function by interpreting a neuronal wiring diagram
Source: Nature. 2024 Oct 2;634(8032):113–23. doi: 10.1038/s41586-024-07953-5 (PMC11446822; doi:10.1038/s41586-024-07953-5)
Supplement: Supplementary file 6 — CRF and ERF predictions for individual TmY4 and TmY9 cells. Analogous to Supplementary Data 3, but for TmY target types. Shown are the top four monosynaptic pathways, the strongest pathway passing through each of the top ten intermediary types (ranking from Extended Data Fig. 7), and the trisynaptic pathway Tm1–TmY–Dm3–TmY (see the section entitled Prediction of spatial normalization). [file 41586_2024_7953_MOESM6_ESM.zip › DataS4/TmY9qΓèÑ/720575940640031576.pdf]

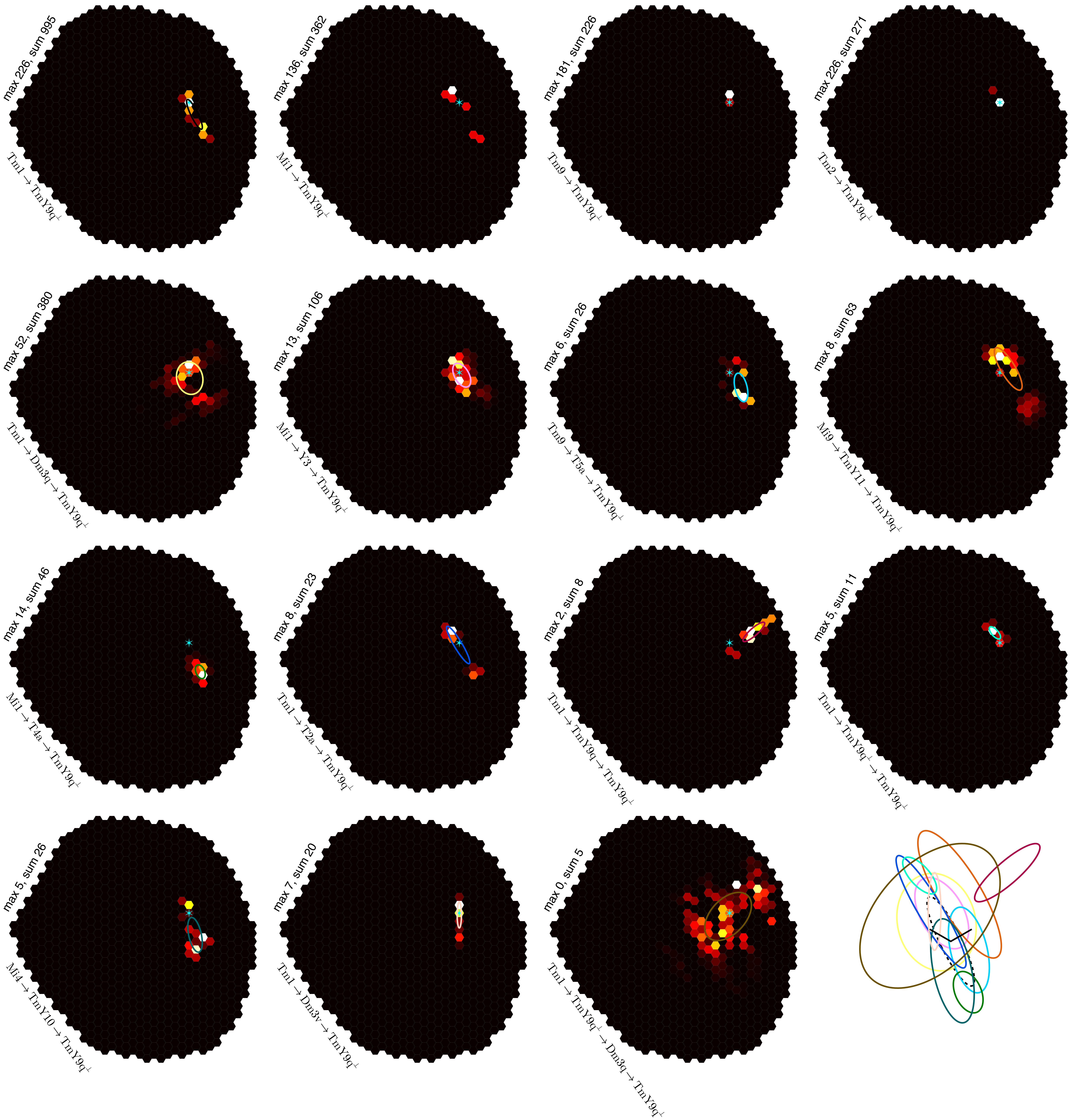
max 226, sum 995
Tm1 → TmY9q⊥
max 136, sum 362
Mi1 → TmY9q⊥
max 181, sum 226
Tm9 → TmY9q⊥
max 226, sum 271
Tm2 → TmY9q⊥
max 52, sum 380
Tm1 → Dm3q → TmY9q⊥
max 13, sum 106
Mi1 → Y3 → TmY9q⊥
max 6, sum 26
Tm9 → T5a → TmY9q⊥
max 8, sum 63
Mi9 → TmY11 → TmY9q⊥
max 14, sum 46
Mi1 → T4a → TmY9q⊥
max 8, sum 23
Tm1 → T2a → TmY9q⊥
max 2, sum 8
Tm1 → TmY9q → TmY9q⊥
max 5, sum 11
Tm1 → TmY9q⊥ → TmY9q⊥
max 5, sum 26
Mi4 → TmY10 → TmY9q⊥
max 7, sum 20
Tm1 → Dm3v → TmY9q⊥
max 0, sum 5
Tm1 → TmY9q⊥ → Dm3q → TmY9q⊥
